# Supplementary figures and images for: Sirt6 deficiency promotes senescence and age-associated intervertebral disc degeneration in mice
Source: Bone Res. 2025 May 8;13:50. doi: 10.1038/s41413-025-00422-3 (PMC12059161; doi:10.1038/s41413-025-00422-3)

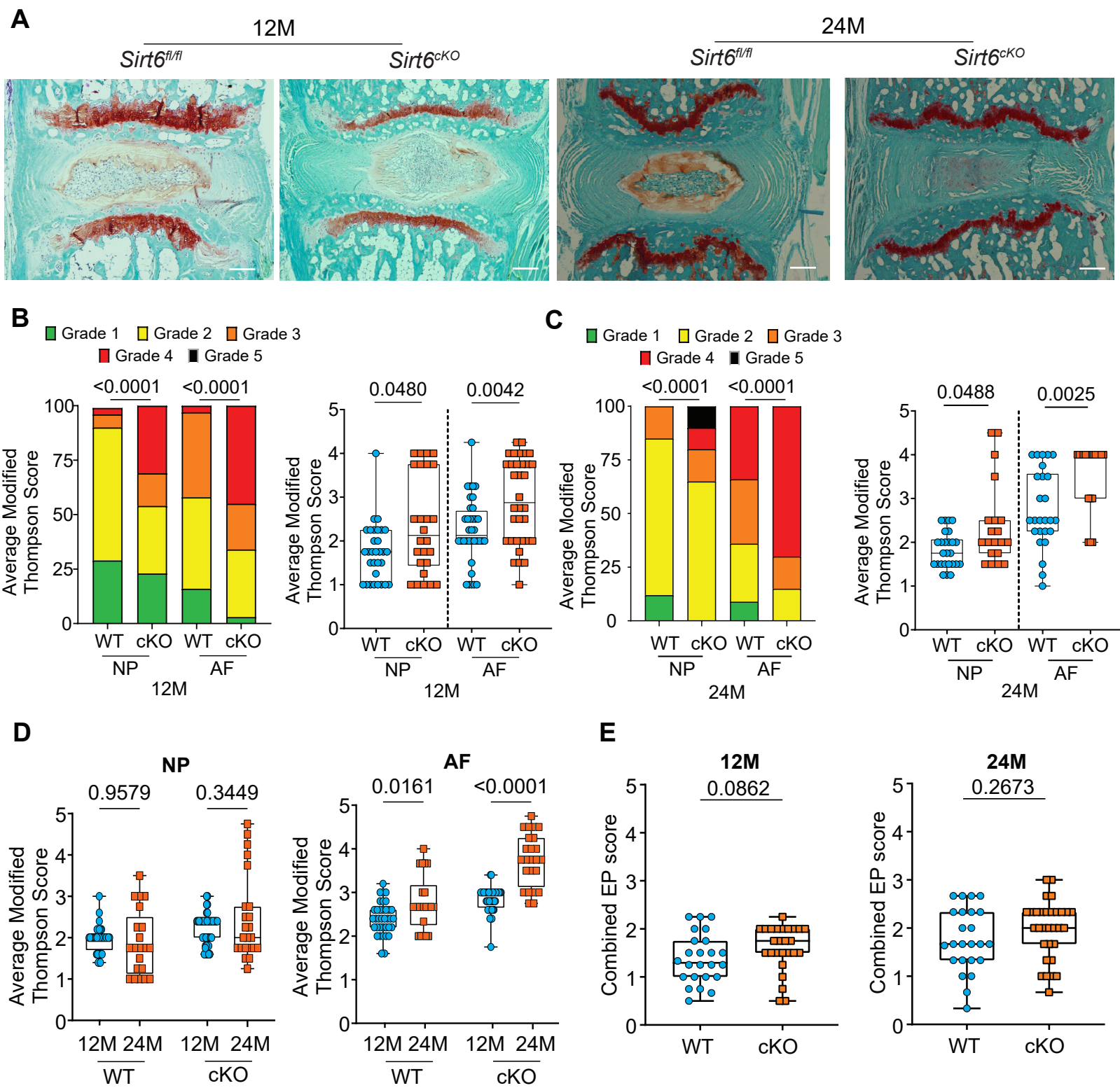

Supplement: Supplementary file 1 — Supplementary Figure 1 [file 41413_2025_422_MOESM1_ESM.pdf]

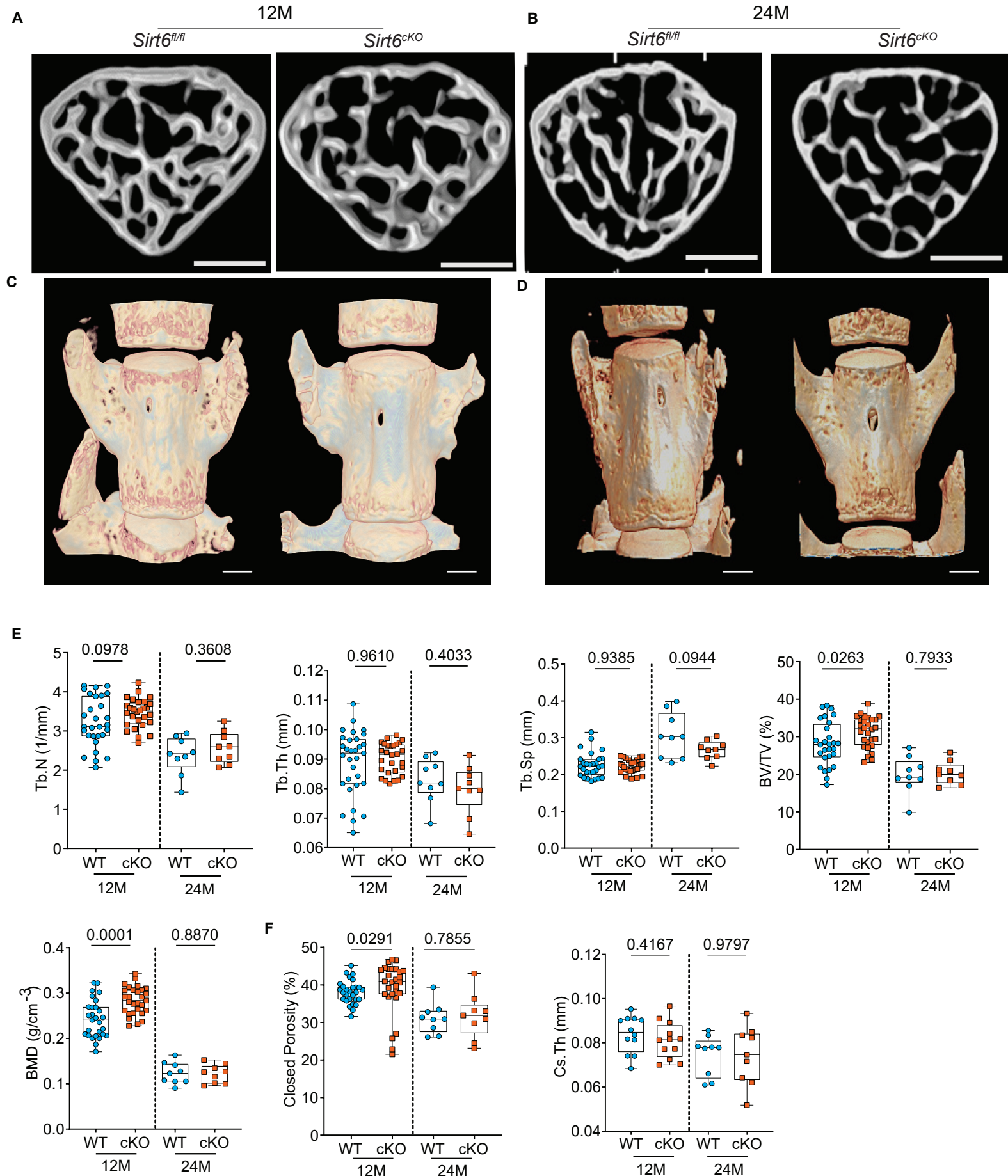

Supplement: Supplementary file 2 — Supplementary Figure 2 [file 41413_2025_422_MOESM2_ESM.pdf]

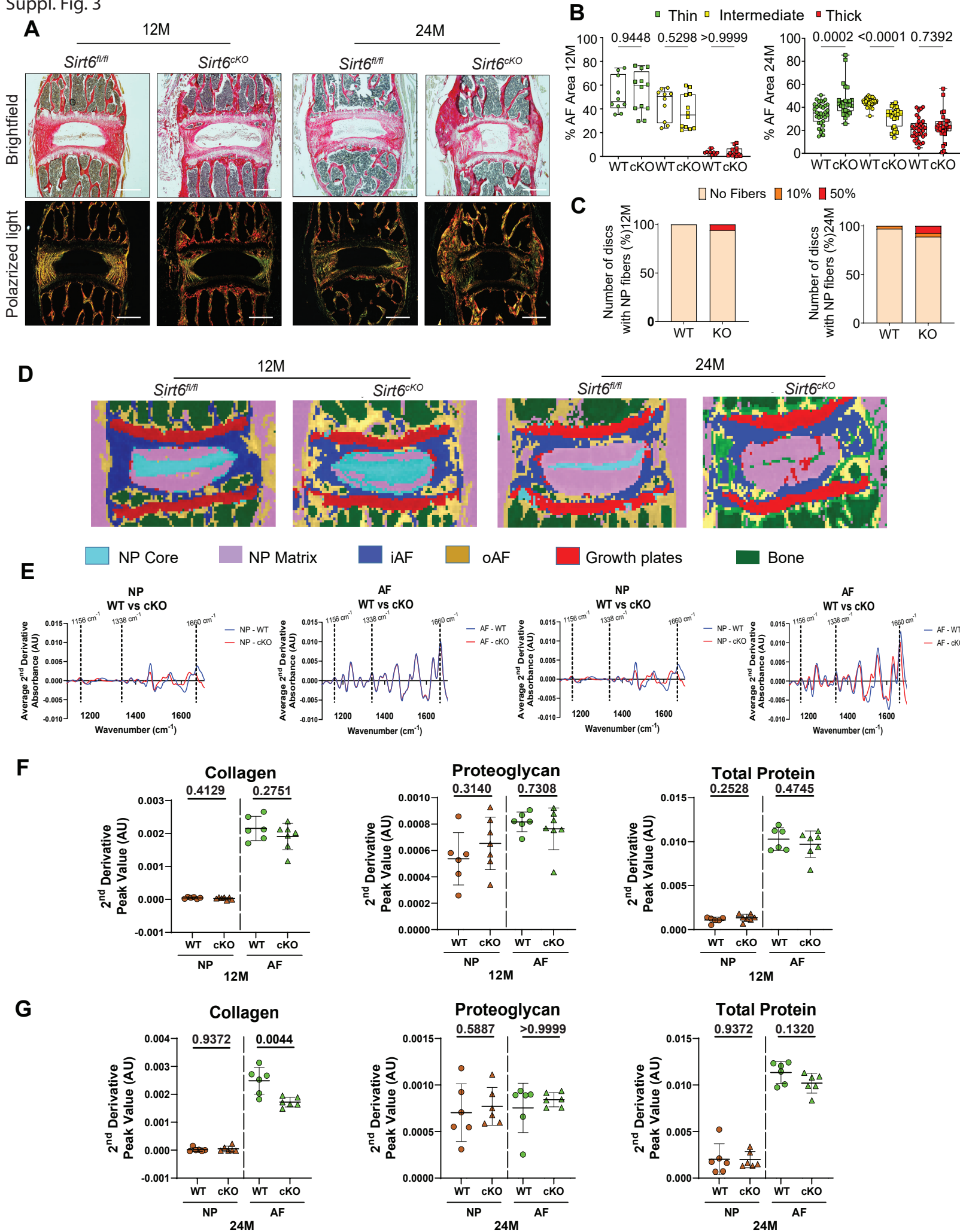

Supplement: Supplementary file 3 — Supplementary Figure 3 [file 41413_2025_422_MOESM3_ESM.pdf]

Suppl. Fig. 4

A PCA Mapping 63.7% (CHP)

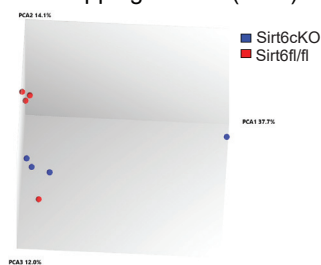

B

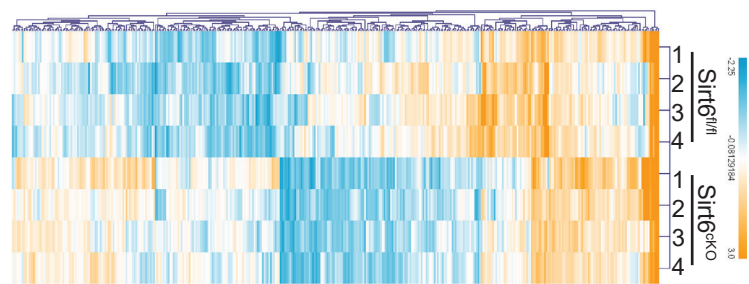

C

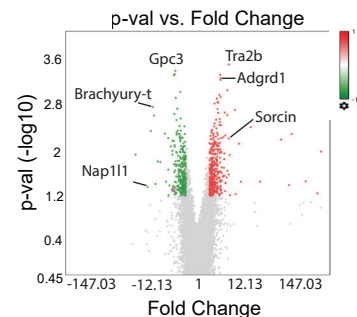

D

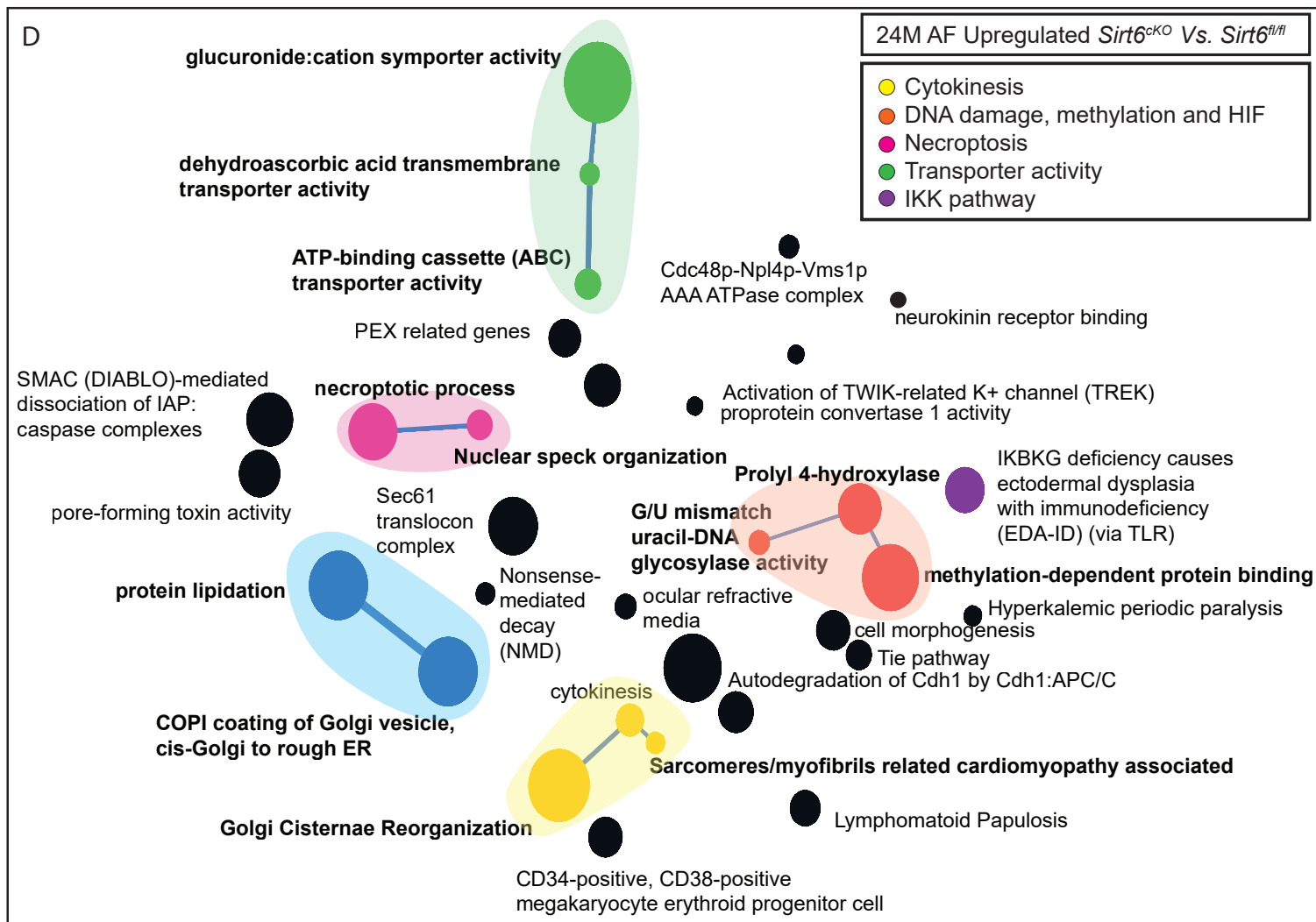

E

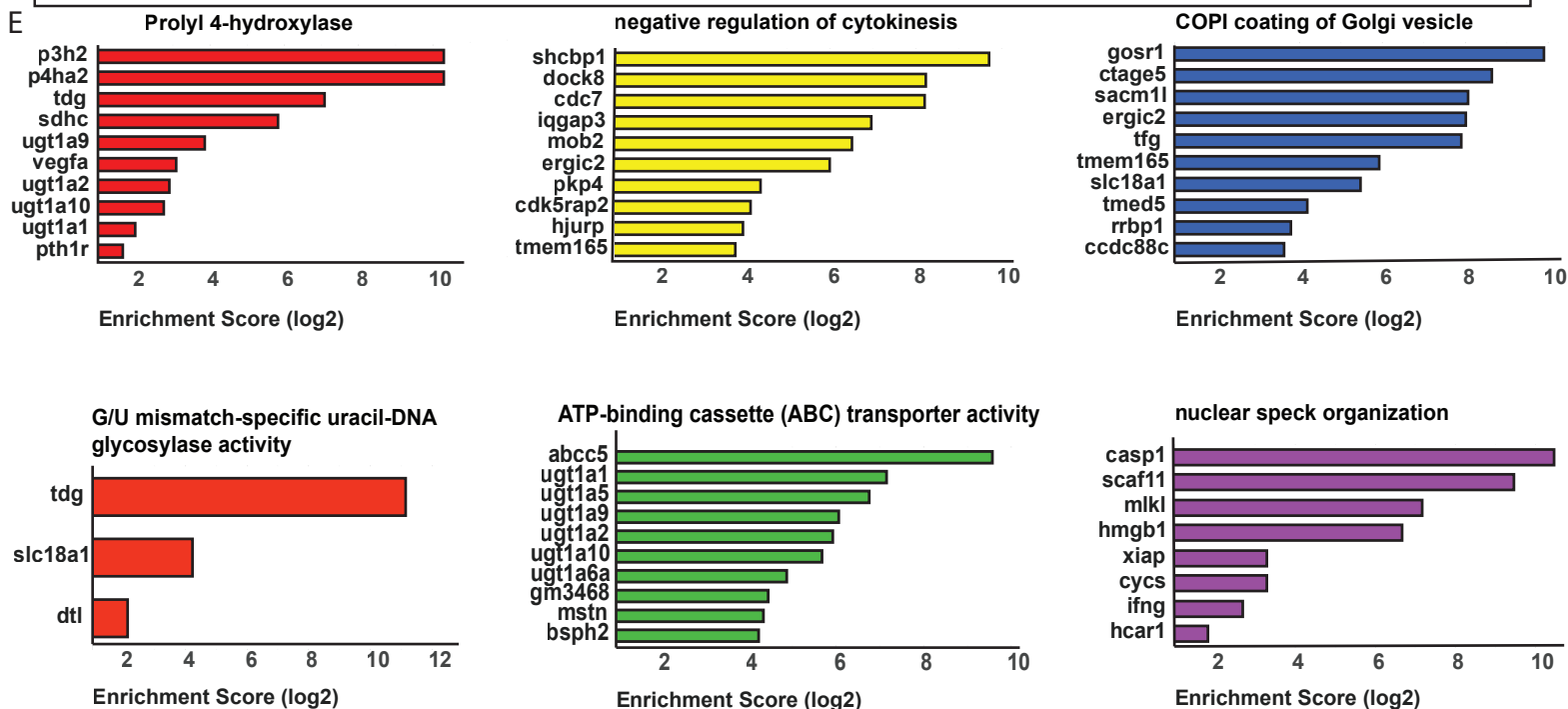

Supplement: Supplementary file 4 — Supplementary Figure 4 [file 41413_2025_422_MOESM4_ESM.pdf]

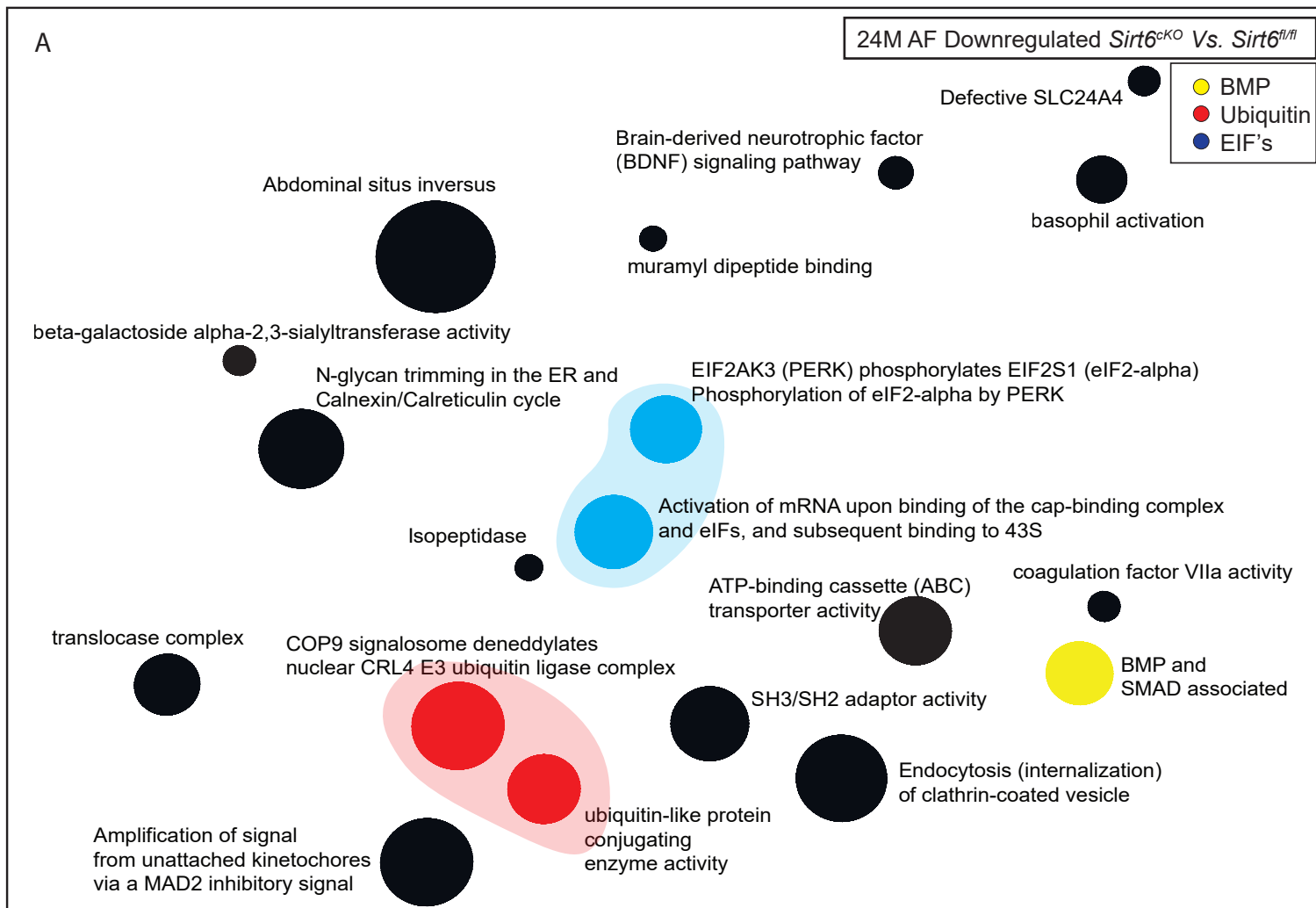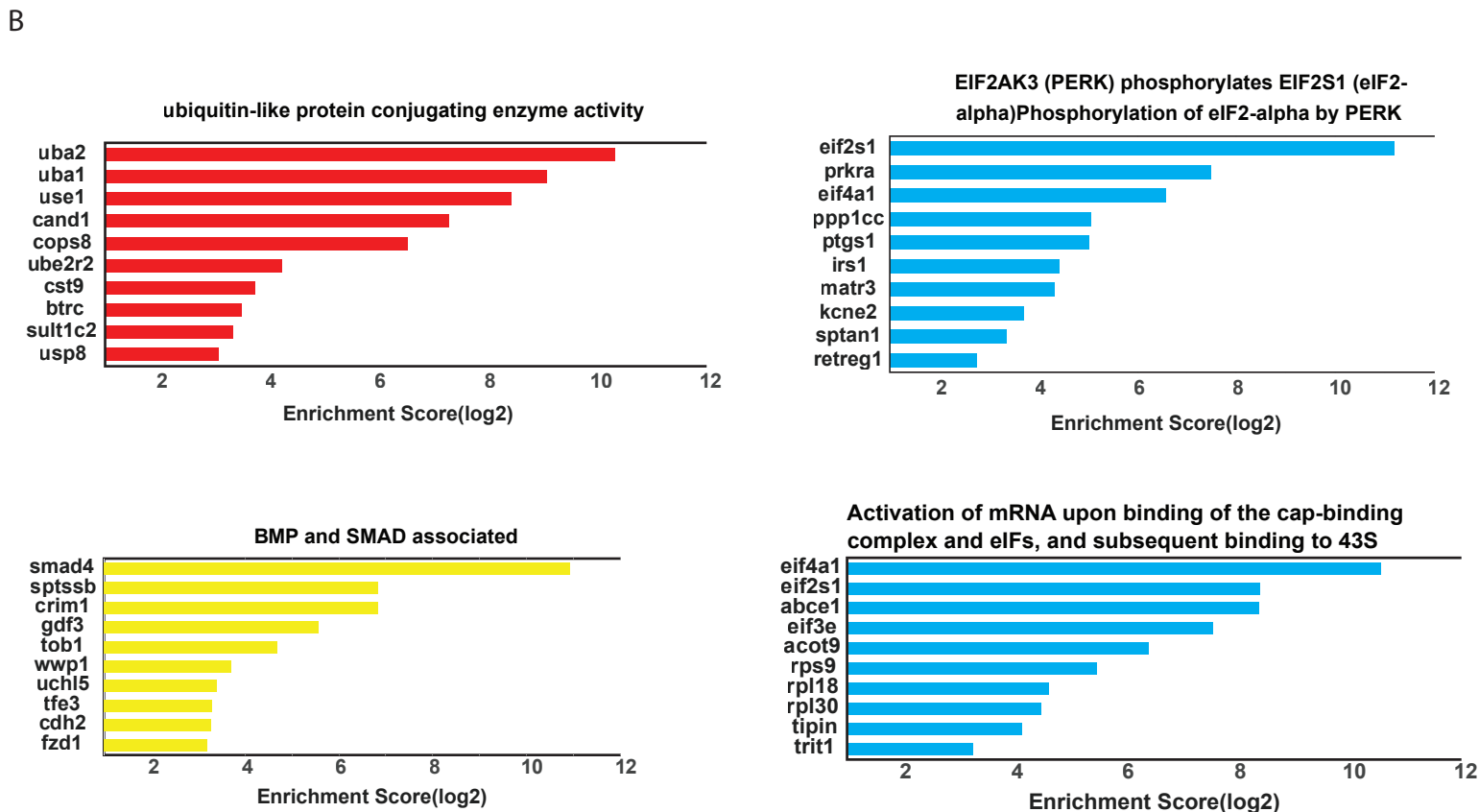

Supplement: Supplementary file 5 — Supplementary Figure 5 [file 41413_2025_422_MOESM5_ESM.pdf]

Suppl. Fig. 6

A

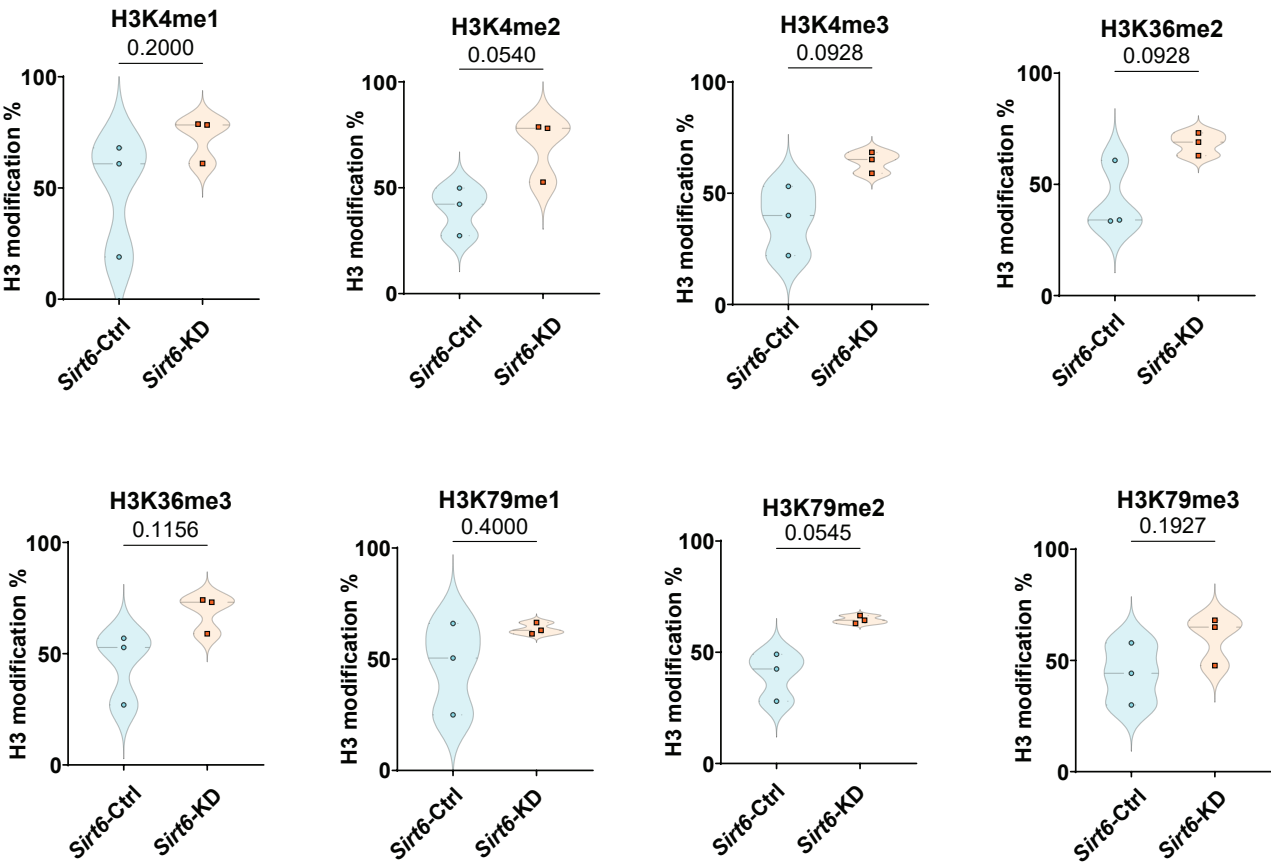

Supplement: Supplementary file 6 — Supplementary Figure 6 [file 41413_2025_422_MOESM6_ESM.pdf]

Suppl. Fig. 7

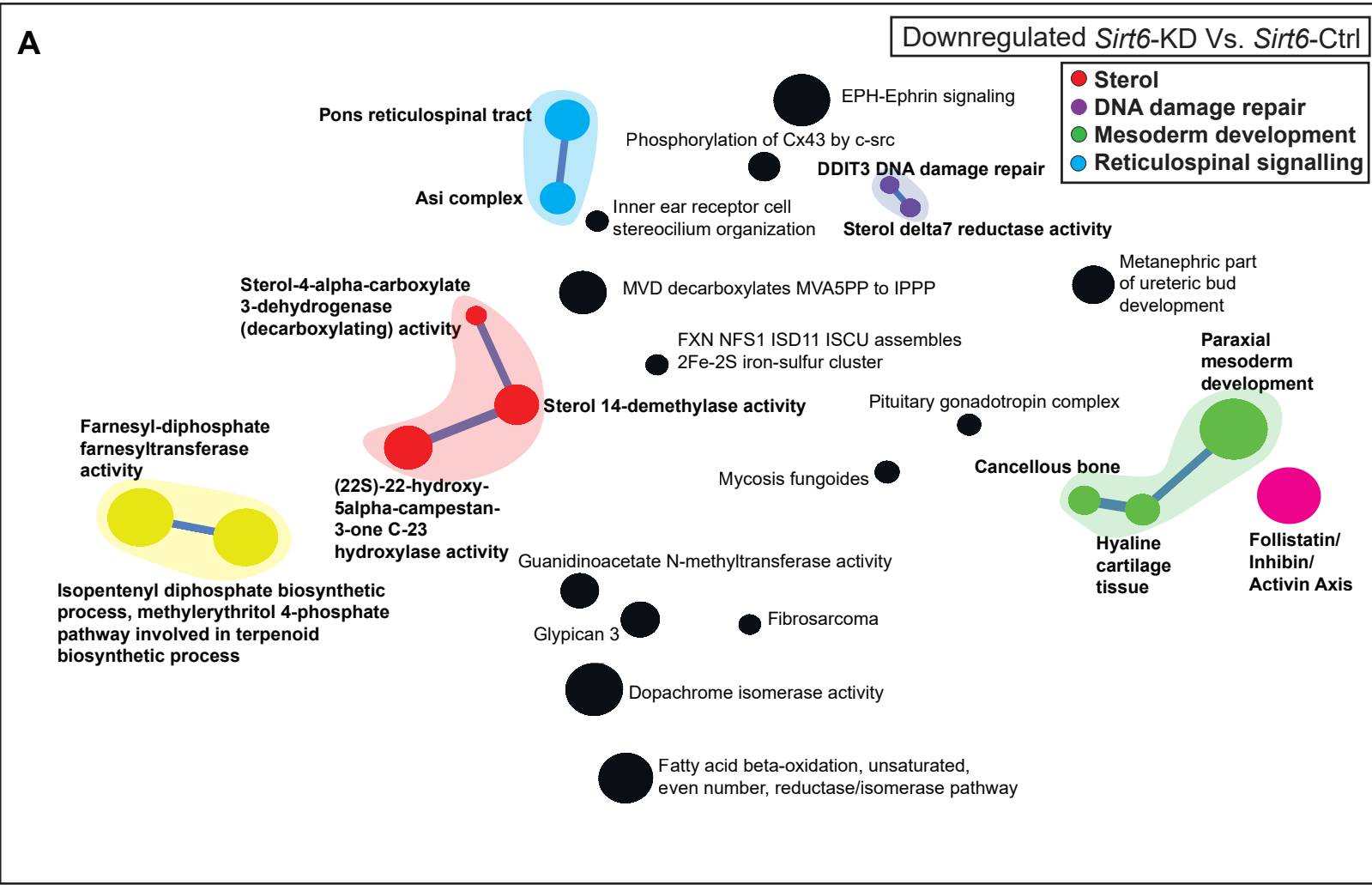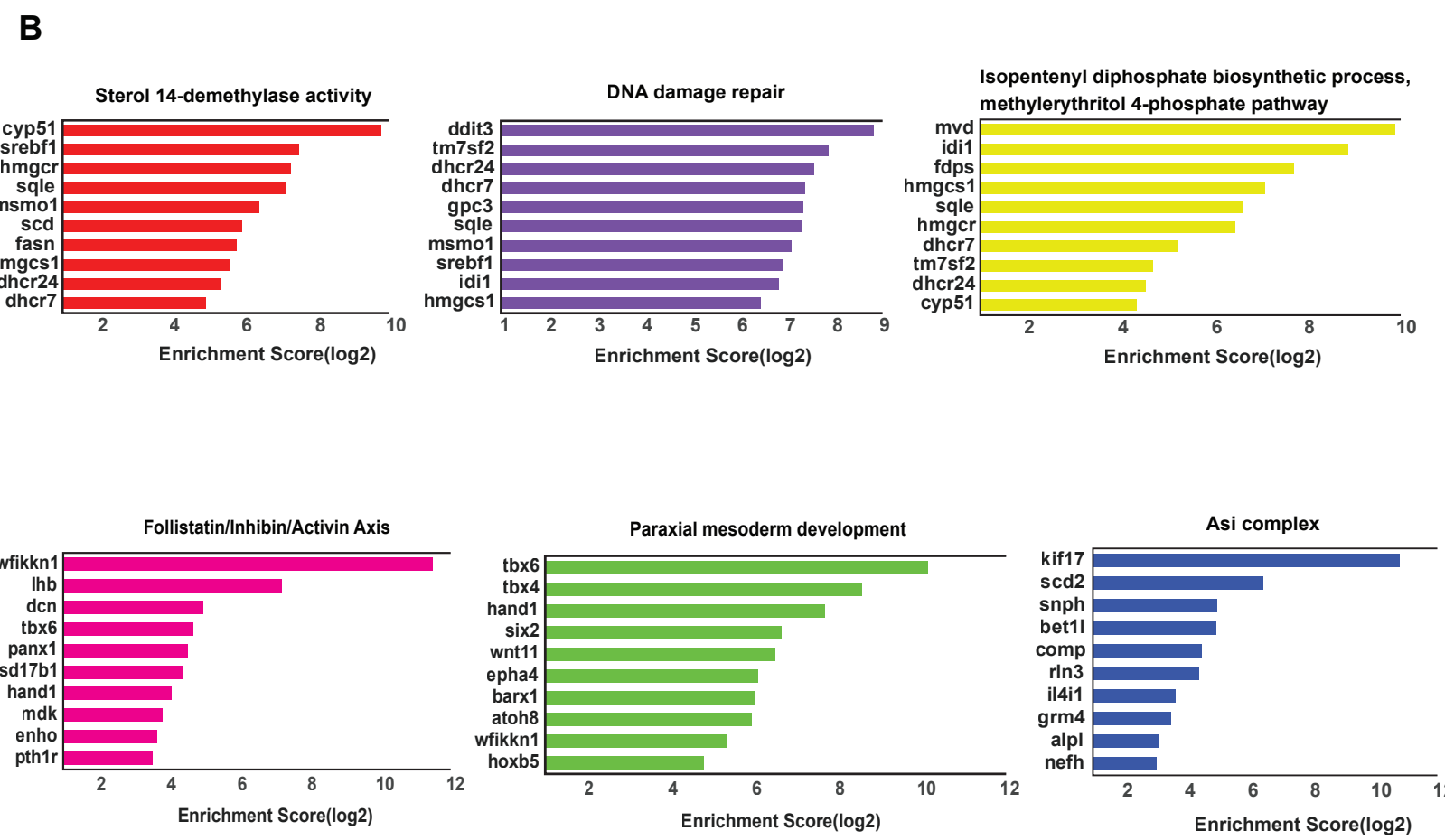

Supplement: Supplementary file 7 — Supplementary Figure 7 [file 41413_2025_422_MOESM7_ESM.pdf]

Suppl. Fig. 8

Upregulated in *Sirt6*<sup>CKO</sup> (NP tissue) Vs. Upregulated in *Sirt6*-KD (NP cells)

A

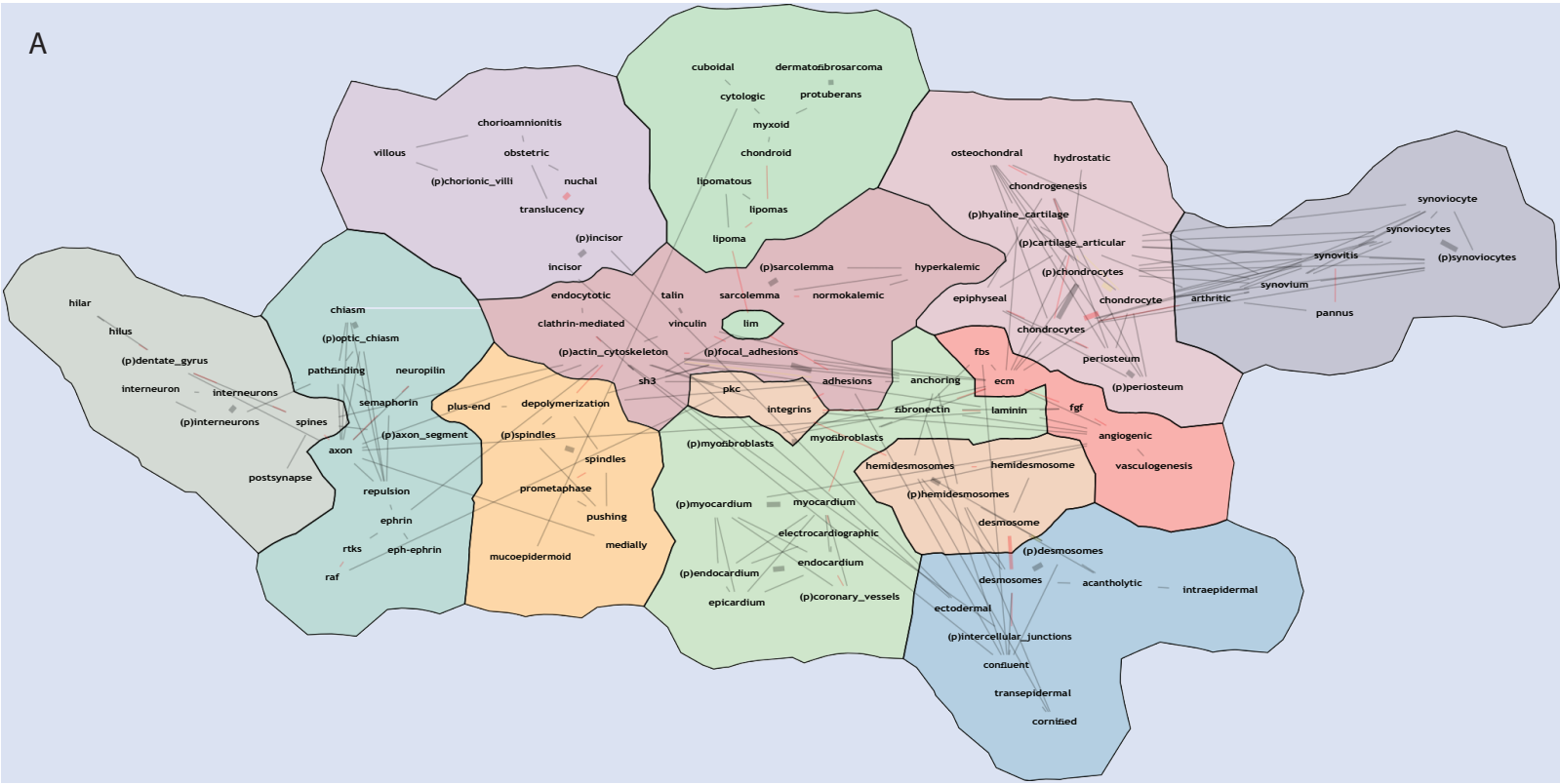

Supplement: Supplementary file 8 — Supplementary Figure 8 [file 41413_2025_422_MOESM8_ESM.pdf]

Suppl. Fig. 9

Downregulated in *Sirt6*<sup>KO</sup> (NP tissue) Vs. Downregulated in *Sirt6*-KD (NP cells)

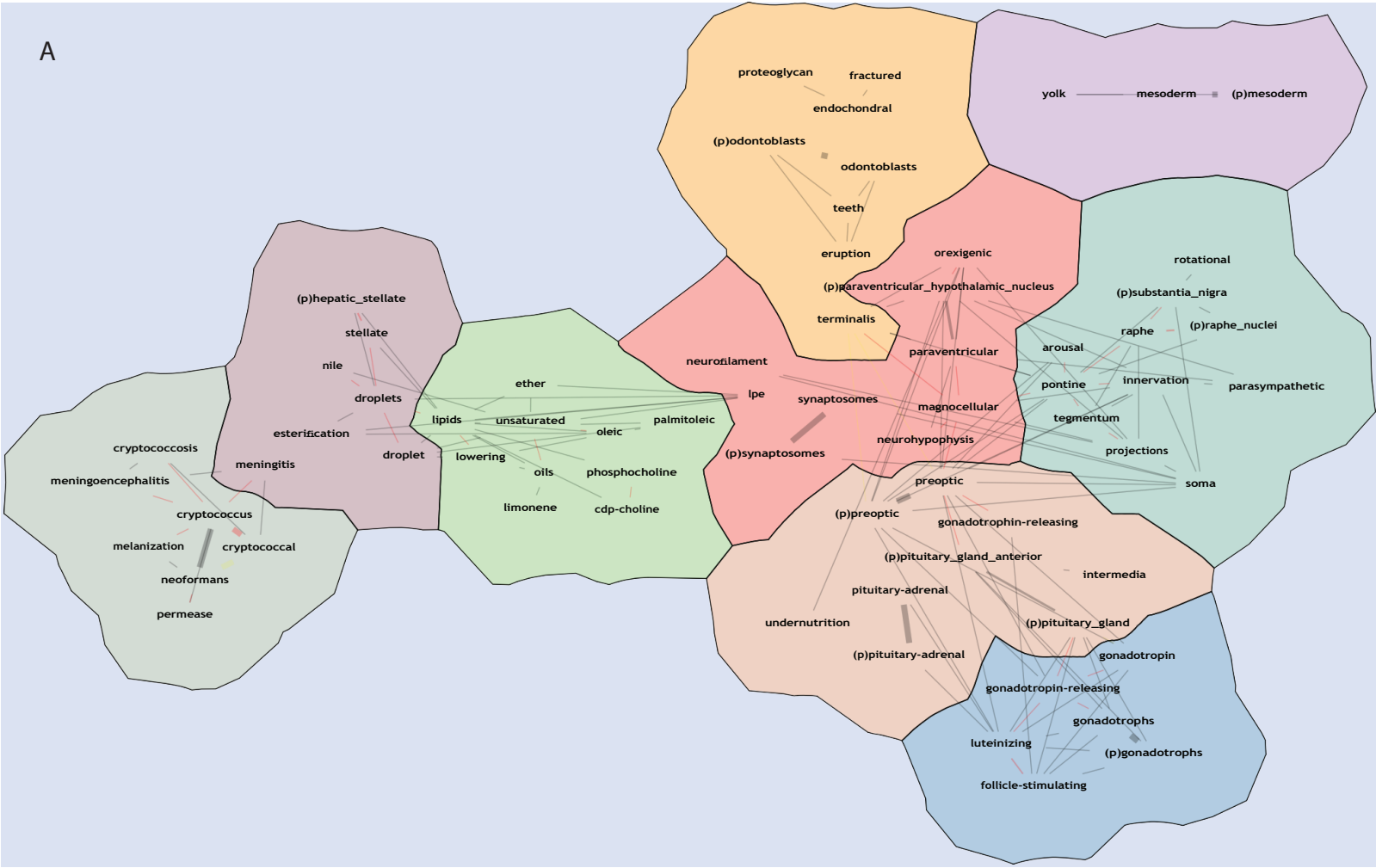

Supplement: Supplementary file 9 — Supplementary Figure 9 [file 41413_2025_422_MOESM9_ESM.pdf]

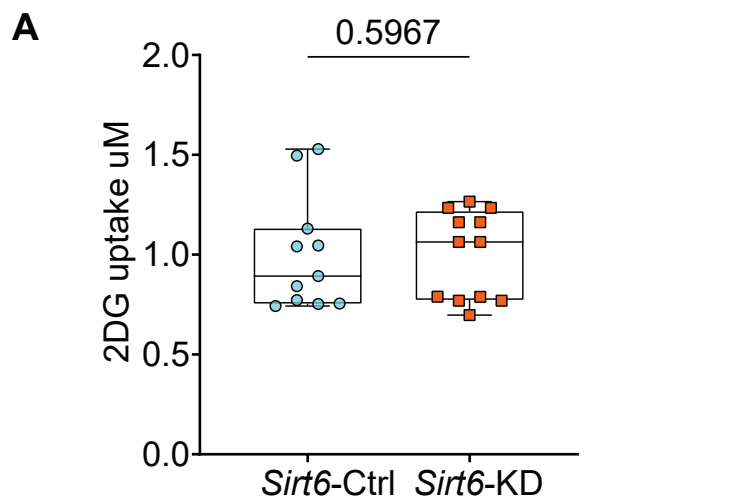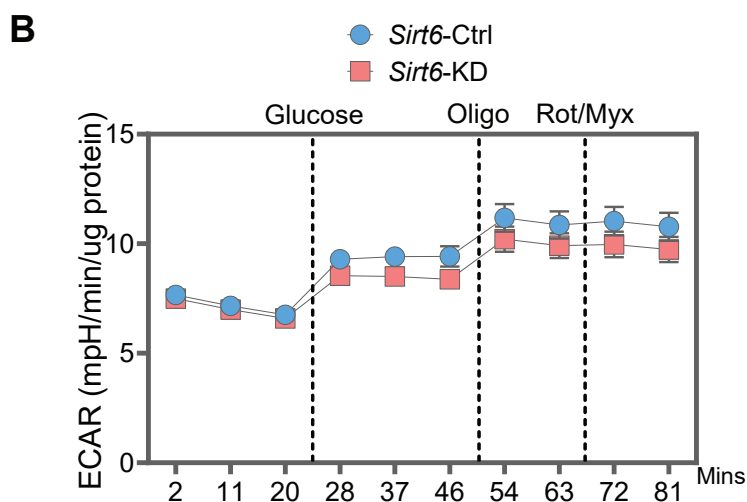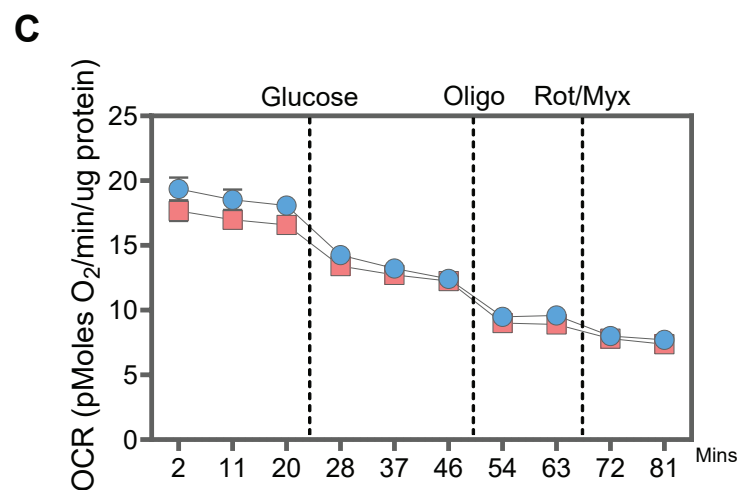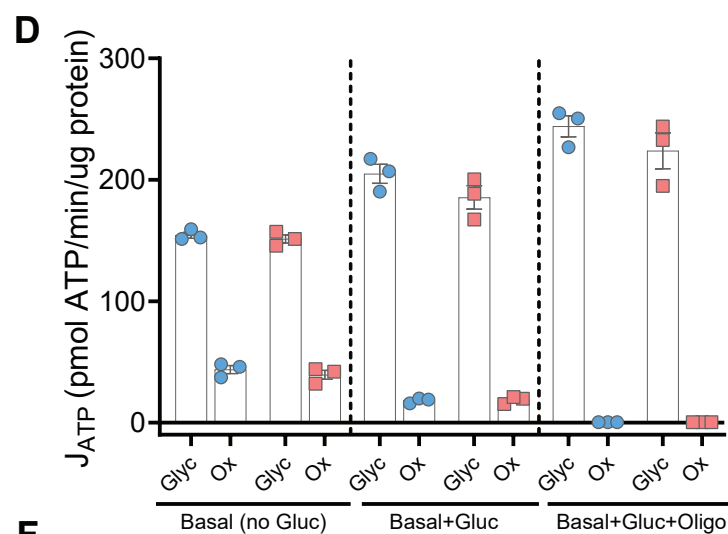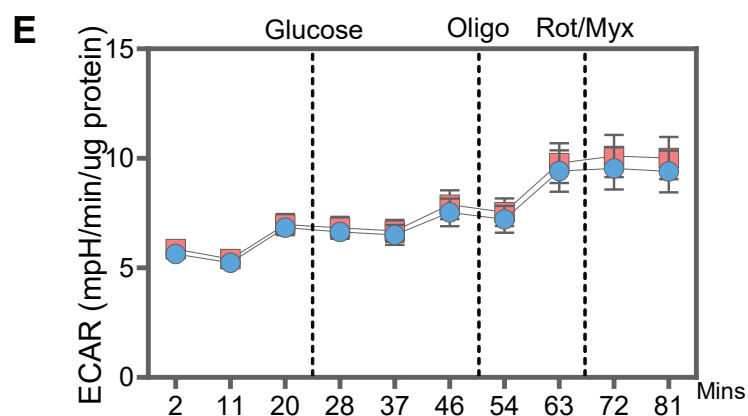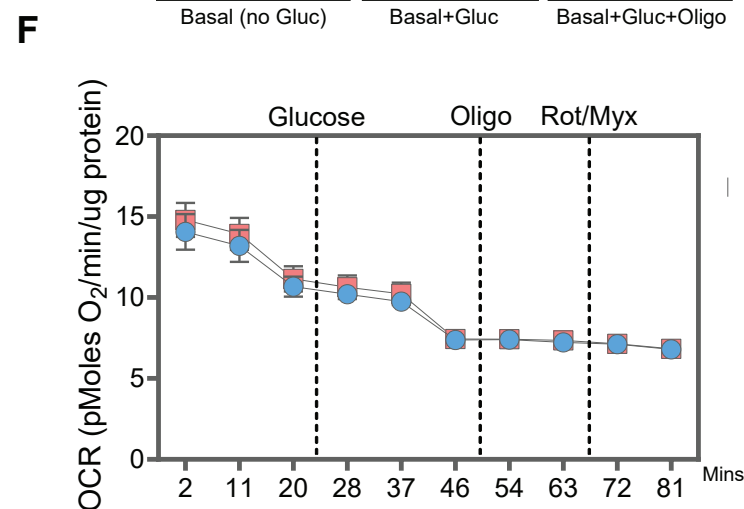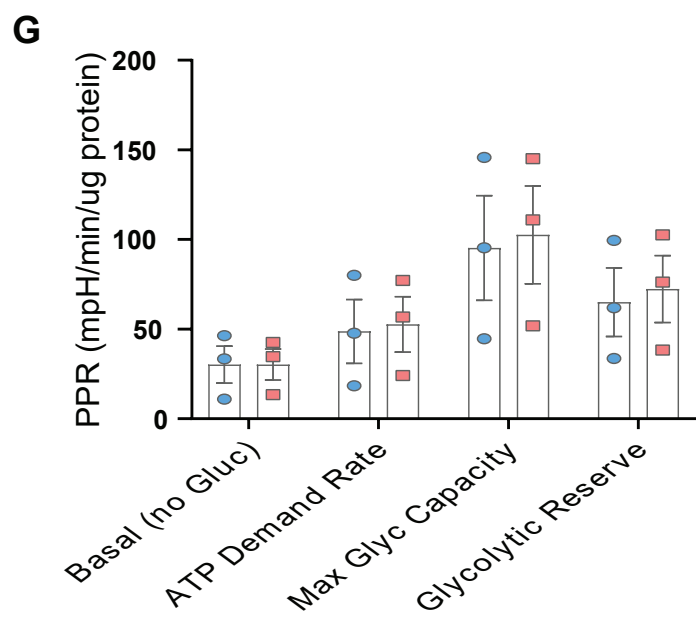

Supplement: Supplementary file 10 — Supplementary Figure 10 [file 41413_2025_422_MOESM10_ESM.pdf]

Upregulated in 24m *Sirt6*<sup>cko</sup> (NP tissue) Vs. Upregulated in 24m *Sirt6*<sup>cko</sup> (AF tissue)

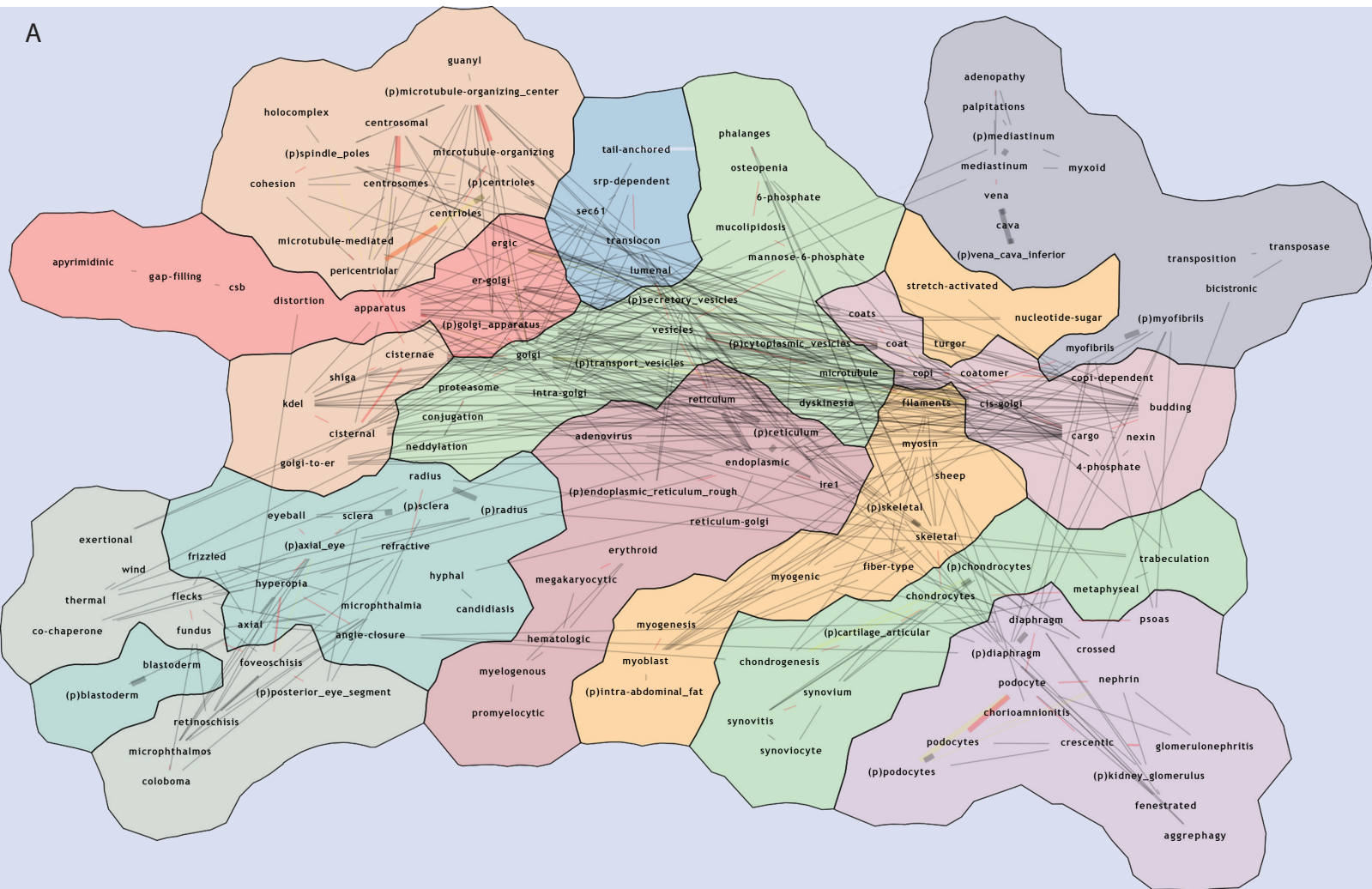

Supplement: Supplementary file 11 — Supplementary Figure 11 [file 41413_2025_422_MOESM11_ESM.pdf]

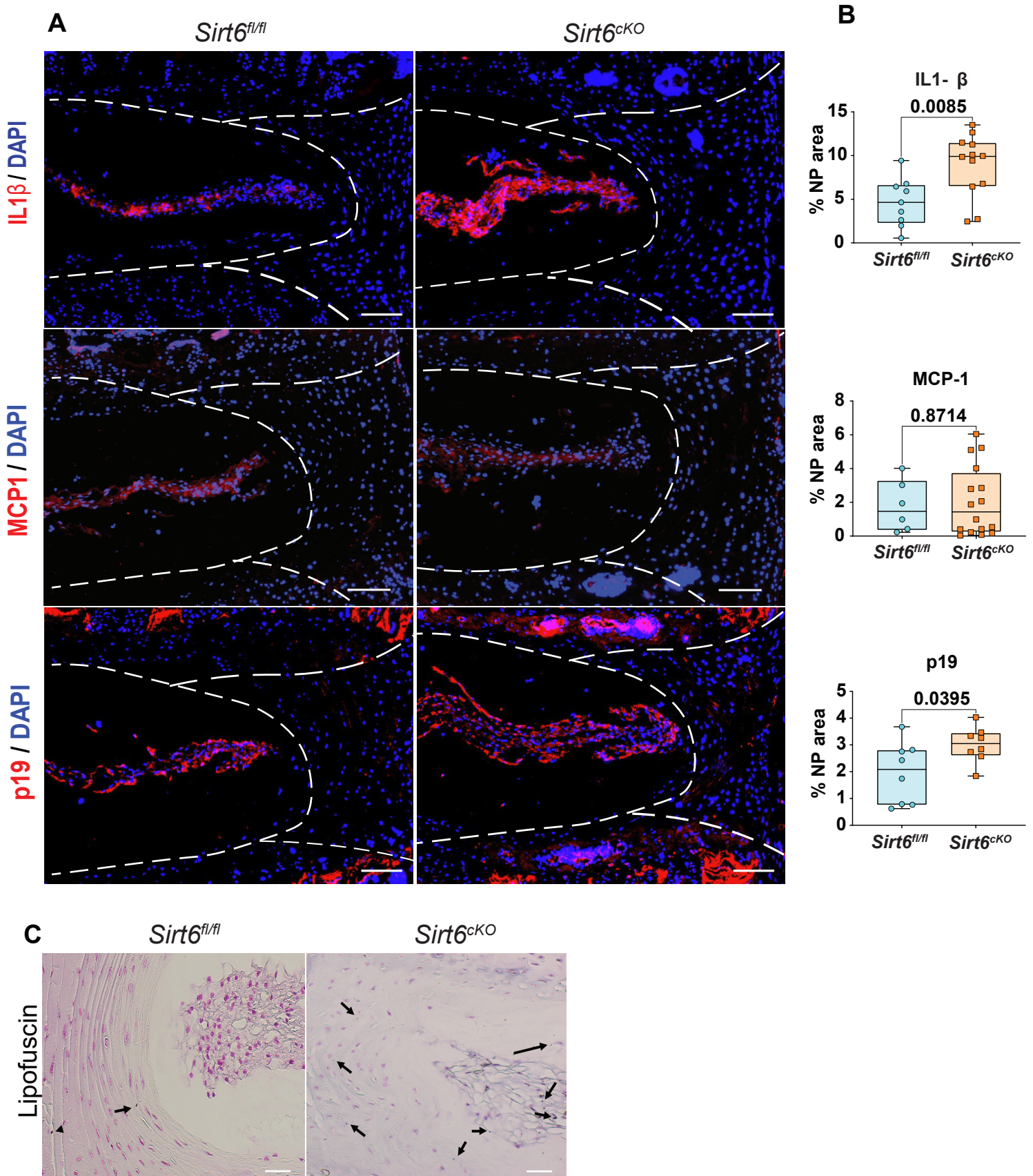

Supplement: Supplementary file 13 — Supplementary Figure 13 [file 41413_2025_422_MOESM13_ESM.pdf]
