# Supplementary material for: Sirt6 deficiency promotes senescence and age-associated intervertebral disc degeneration in mice
Source: Bone Res. 2025 May 8;13:50. doi: 10.1038/s41413-025-00422-3 (PMC12059161; doi:10.1038/s41413-025-00422-3)

Suppl. Fig. 12

Downregulated in *Sirt6*cKO (NP tissue) Vs. Downregulated in *Sirt6*cKO (AF tissue)

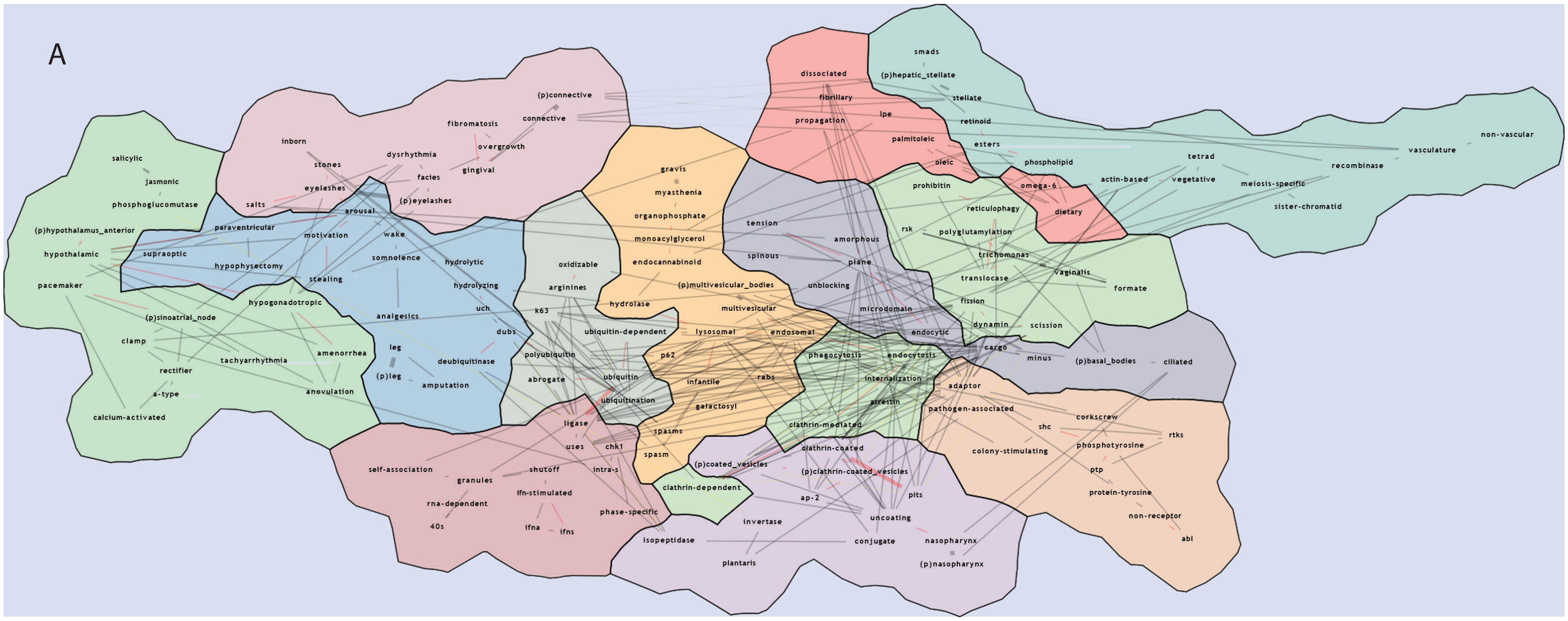

Supplement: Supplementary file 12 — Supplementary Figure 12 [file 41413_2025_422_MOESM12_ESM.pdf]
