# Supplementary material for: Sirt6 deficiency promotes senescence and age-associated intervertebral disc degeneration in mice
Source: Bone Res. 2025 May 8;13:50. doi: 10.1038/s41413-025-00422-3 (PMC12059161; doi:10.1038/s41413-025-00422-3)
Supplement: Supplementary file 14 — Supplementary Figure Legends [file 41413_2025_422_MOESM14_ESM.docx]

**Supplemental Figure Legends:**

**Supplementary Figure 1:** (A) Representative images of Safranin-O/Fast Green staining of *Sirt6*^cKO^ and *Sirt6*^fl/fl^ caudal discs at 12 months and 24 months. Scale bar = 200 μm. (B-C) Distribution and Average Modified Thompson Grades of caudal discs of *Sirt6*^cKO^ and *Sirt6*^fl/fl^ at (B) 12 months and (C) 24 months. N = 3-6 mice/genotype, 3-4 discs/animal. (D) Age dependency of Modified Thompson Grades for NP and AF. (E) ORS Spine Section Grading scores for endplates of *Sirt6*^cKO^ and *Sirt6*^fl/fl^ caudal discs at 12 and 24 months. N = 4-6 animals/genotype and 3-4 levels/animal. Statistical difference between grade distributions was tested using chi-square test, all other quantitative data was compared using unpaired t-test, *p < 0.05.

**Supplementary Figure 2:** Representative μCT reconstructions showing (A-B) transverse section through lumbar vertebra and (C-D) lumbar motion segment in 12M and 24M old *Sirt6*^fl/fl^ and *Sirt6*^cKO^ mice. (E) Trabecular bone properties, BV/TV, Tb. Th. (trabecular thickness), Tb. N. (trabecular number), Trab. Sp. (trabecular separation) (F) Cortical bone properties of Cs. Th. (cross-sectional thickness) and closed porosity are shown. Statistical difference between groups was tested using unpaired t-test, *p < 0.05.

**Suppl. Figure 3:** (A) Representative polarized images of Picrosirius Red-stained lumbar disc sections and (B) quantification of collagen fibers from 12 and 24M *Sirt6*^fl/fl^ and *Sirt6*^cKO^ mouse discs. n= 4-6 animals/genotype and 3-4 discs/animal. Scale bar= 200 μM. (C) Quantification of NP compartment fibrosis. (D) Spectral cluster analysis images of 12 M and 24 M discs (scale bar = 200 μm). IVD n=4-6 animals/genotype and 3-4 discs/animal. Scale bar=200uM. (E) Average superimposed second derivative spectra, inverted for positive visualization of the NP and AF of 12M and 24M old mice. (F, G) quantification of mean second derivative peaks for 12M (F) and 24M (G). Significance for quantitative measures was determined by using an unpaired *t*-test with Mann–Whitney test or Welch’s correction, as appropriate. Quantitative measurements represent the median with the interquartile range.

**Supplementary Figure 4**: Microarray analysis of AF tissue transcripts from *Sirt6*^fl/fl^ and *Sirt6*^cKO^ represented as (A) Three-dimensional Principal component analysis (PCA) showing discrete clustering of based on genotype (*n* = 4 mice/genotype, 5-6 pooled discs/animal) (B) Heat map and hierarchical clustering of Z-score of differentially expressed genes (DEGs) between *Sirt6^fl^*^/fl^ and *Sirt6*^cKO^ (*p* ≤ 0.05, FC≥1.75). (C) Volcano plot of DEGs in the AF showing *p*-value *versus* magnitude of change (fold change).  (D) CompBIO analysis of Upregulated DEGs in AF tissue of 24M *Sirt6*^cKO^ represented in a ball and stick model. The enrichment of themes is shown by the size of the ball and connectedness is shown based on thickness of the lines between them. Themes of interest are colored, and superclusters comprised of related themes are highlighted. (E) Top thematic DEGs plotted based on CompBio entity enrichment score.

**Supplementary Figure 5**: (A) CompBIO analysis of downregulated DEGs in AF tissue of from 24M *Sirt6*^cKO^ mice represented as a ball and stick model (FC>1.75, p>0.05). n=4 mice/genotype 5-6 pooled discs/animal. The enrichment of themes is shown by the size of the ball and connectedness is shown based on thickness of the lines between them. Themes of interest are colored, and superclusters comprised of related themes are highlighted. (B) Top thematic DEGs plotted based on CompBio entity enrichment score.

**Supplementary Figure 6:** (A) Quantitative ELISA showing Histone 3 modifications in *Sirt6*-KD compared to *Sirt6*-Ctrl NP cells (n=3 independent experiments). Significance for quantitative measures was determined by using an unpaired *t*-test.

**Supplementary Figure 7:** (A) Compbio analysis for downregulated DEGs from RNAseq analysis of *Sirt6*-KD Vs. *Sirt6*-Ctrl NP cells (FC>1.5, FDR<0.05), represented as a ball and stick model. The enrichment of themes is shown by the size of the ball and connectedness is shown based on thickness of the lines between them. Themes of interest are colored, and superclusters comprised of related themes are highlighted. (B) Top thematic DEGs plotted based on CompBio entity enrichment score.

**Supplementary Figure 8**: Territorial maps of CompBio Assertion engine analysis showing shared themes between upregulated DEGs from NP tissues of *Sirt6^cKO^* vs *Sirt6*-KD NP cells, p>0.05.

**Supplementary Figure 9:** Territorial maps of CompBio Assertion engine analysis showing shared themes between downregulated DEGs from NP tissues of *Sirt6*^cKO^ vs *Sirt6*-KD NP cells, p>0.05.

**Supplementary Figure 10**: (A) 2-Deoxyglucose (2-DG) uptake in *Sirt6*-Ctrl Vs. Sirt6-KD NP cells for 24 hours. (B-D) OCR and ECAR traces in *Sirt6*-Ctrl Vs. Sirt6-KD NP cells for 24-hour to assess ATP production rate. (E-G) OCR and ECAR traces in *Sirt6*-Ctrl Vs. *Sirt6*-KD NP cells for 24-hour to assess proton production rate.

**Supplementary Figure 11**: (A) Territorial maps of CompBio Assertion engine analysis showing shared themes between upregulated DEGs from NP vs. AF tissues of *Sirt6^cKO^*, p>0.05.

**Supplementary Figure 12**: (A) Territorial maps of CompBio Assertion engine analysis showing shared themes between downregulated DEGs from NP vs. AF tissues of *Sirt6^cKO^*, p>0.05.

**Supplementary Figure 13**: (A) Representative immunofluorescence images and (B) quantitative analysis of SASP markers IL-1β, MCP1, and p19. n= 5-8 animals/genotype, 1-3 discs/mouse. Scale bar = 50 μM (C) Representative images of Sudan Black staining of intervertebral discs show increased Lipofuscin accumulation in *Sirt6*^cKO^ compared to *Sirt6*^fl/fl^.
